# Audio-Based Engine Fault Diagnosis with Wavelet, Markov Blanket, ROCKET, and Optimized Machine Learning Classifiers

**DOI:** 10.3390/s24227316

**Published:** 2024-11-15

**Authors:** Bernardo Luis Tuleski, Cristina Keiko Yamaguchi, Stefano Frizzo Stefenon, Leandro dos Santos Coelho, Viviana Cocco Mariani

**Affiliations:** 1 Department of Mechanical Engineering, Pontifical Catholic University of Parana, Curitiba 80242-980, PR, Brazil; bernardo_t@live.com; 2 Robert Bosch Ltda, Av. Juscelino Kubitschek de Oliveira, 11800, Cidade Industrial, Curitiba 80242-980, PR, Brazil; 3 Postgraduate Program in Productive Systems in Association with UNIPLAC, UNC, UNESC, and UNIVILLE, Lages 88509-900, SC, Brazil; criskyamaguchi@gmail.com; 4 Graduate Program in Electrical Engineering, Federal University of Parana, Curitiba 80242-980, PR, Brazil; leandro.coelho@ufpr.br; 5 Department of Electrical Engineering, Federal University of Parana, Curitiba 80242-980, PR, Brazil; viviana.mariani@ufpr.br; 6 Graduate Program in Mechanical Engineering, Federal University of Parana, Curitiba 80242-980, PR, Brazil

**Keywords:** machine learning classifiers, Markov blanket, random convolutional kernel transform (ROCKET), time-series classification, wavelet packet transform

## Abstract

Engine fault diagnosis is a critical task in automotive aftermarket management. Developing appropriate fault-labeled datasets can be challenging due to nonlinearity variations and divergence in feature distribution among different engine kinds or operating scenarios. To solve this task, this study experimentally measures audio emission signals from compression ignition engines in different vehicles, simulating injector failures, intake hose failures, and absence of failures. Based on these faults, a hybrid approach is applied to classify different conditions that help the planning and decision-making of the automobile industry. The proposed hybrid approach combines the wavelet packet transform (WPT), Markov blanket feature selection, random convolutional kernel transform (ROCKET), tree-structured Parzen estimator (TPE) for hyperparameters tuning, and ten machine learning (ML) classifiers, such as ridge regression, quadratic discriminant analysis (QDA), naive Bayes, *k*-nearest neighbors (*k*-NN), support vector machine (SVM), multilayer perceptron (MLP), random forest (RF), extra trees (ET), gradient boosting machine (GBM), and LightGBM. The audio data are broken down into sub-time series with various frequencies and resolutions using the WPT. These data are subsequently utilized as input for obtaining an informative feature subset using a Markov blanket-based selection method. This feature subset is then fed into the ROCKET method, which is paired with ML classifiers, and tuned using Optuna using the TPE approach. The generalization performance applying the proposed hybrid approach outperforms other standard ML classifiers.

## 1. Introduction

The worldwide automotive aftermarket industry was estimated to be worth 342 million dollars in 2022. According to projections, the market encompassing light, medium, and heavy-duty vehicles is expected to grow significantly, with a compound annual growth rate of 5.5%. This expansion points to an important future for the automotive aftermarket industry. Currently, vehicle part suppliers contribute significantly to this industry, manufacturing 77% of the value found in contemporary automobiles. Looking ahead to 2030, a transformative shift is anticipated as an estimated 95% of newly sold vehicles worldwide are expected to be connected. This projection underscores the increasing integration of technology and connectivity within the automotive sector [1].

In the automotive aftermarket industry, visual inspections and sensor data have traditionally played a significant part in our diagnostic capacity to assess, for example, the condition of internal combustion engines [2]. However, as current engines become more complex, we will need to use more current techniques for the diagnostic of these signals [3]. Engineers and researchers can use the diverse noises made by engines at various stages of operation as an audible fingerprint to detect problems such as ignition faults [4], irregular valve timing, and even approaching component failures [5]. Using audio categorization adds a new level of diagnostic capacity and provides a real-time, non-intrusive method to check engine health [6].

Although automotive engine ignition systems are built differently, they always follow the same basic principles of operation. Every system has a primary circuit that causes the secondary circuit to spark. The next step is to transfer this spark to the appropriate spark plug at the exact moment. Conditions in the cylinder and ignition system impact the secondary circuit’s ignition pattern, also known as the scope pattern. A scope meter, often called an automotive oscilloscope, is a useful tool for diagnosing engine and ignition problems. It does this by displaying scope patterns that make it possible to conduct a thorough examination of the ignition system’s operation [7].

Regarding the detection of faults in internal combustion engines in automobiles, it should be noted that the process of capturing these ignition patterns often involves manual intervention by mechanics [8]. Subsequently, the captured pattern is compared with reference samples from handbooks to facilitate diagnosis. However, this diagnostic process heavily relies on domain knowledge and user experience since handbook samples serve as references only. The challenge arises from the fact that ignition patterns are dynamic and non-stationary time series [9], varying in amplitude and duration across different engine models experiencing the same ignition system trouble. Even within the same engine, different patterns may happen over distinct operating conditions, confounding diagnosis. Additionally, many engine faults manifest similar ignition patterns, further making it difficult the accurate identification of issues [10].

Mechanics expend significant time and effort due to the inherent inaccuracies in human diagnosis and the necessity for multiple trials involving the disassembling and assembling of engine parts. To address this, our proposed solution involves the generation of a ML-based pattern classification system, aimed at assisting automotive mechanics. ML models could analyze and interpret the acoustic signals produced during engine operation in the context of internal combustion engines for audio classification. Such algorithms can be designed to identify between multiple engine states, detecting anomalies, probable failures, and normal operation [11].

To extract meaningful information from the complex auditory landscape of engines, researchers employ methodologies such as spectrum analysis, frequency domain approaches, and deep learning models. These acoustic data and artificial intelligence not only enhanced the understanding of engine behavior but also helped to make more informed decisions in terms of maintenance, efficiency optimization, and overall performance. While there are many potential benefits to audio classification in internal combustion engines, there are also challenges to the field. The inherently noisy environment of an operating engine poses difficulties in isolating specific sounds and patterns [12].

Variations in engine designs, fuel types, and operating conditions add complexity to the task of developing robust and generalized classification models. Overcoming these challenges requires a multimodel approach, combining acoustics, signal processing, and ML to generate efficient solutions The combination of audio categorization of internal combustion engines offers a significant advancement in the search for a better understanding of vehicle systems. Based on these aspects, the use of acoustics and artificial intelligence has the potential to revolutionize the future of vehicle maintenance [13].

Compression ignition engines have become known as both reliable and efficient. However, like any complex device, engines are subject to various failures that can affect their performance and durability. For example, mechanical failures can include problems with the piston, connecting rod, crankshaft, valves, and piston rings, among other internal components. These faults can result in abnormal vibrations, metallic noises, or engine knocking. Another example of failure could be injector problems, such as clogging, leakage, or malfunction, which can lead to inadequate performance, including lack of power and altered emissions [14].

Selecting the most appropriate classifier can be challenging, as each model can perform better depending on its application [15]. To obtain the best possible result, several state-of-the-art classifiers are considered in this paper. The classifier is defined by the best performance result achieved by the model, considering the use of the optimized hyperparameters, thus ensuring that an adequate model is obtained for the proposed task.

Klaar et al. [16] integrates ROCKET with ML classifiers and empirical mode decomposition techniques like Complete Ensemble Empirical Mode Decomposition with Adaptive Noise (CEEMDAN), Empirical Wavelet Transform (EWT), and Variational Mode Decomposition (VMD). Results demonstrate the accuracy improvement achieved by combining these methods, with accuracies of 0.992 using CEEMDAN, 0.995 using EWT, and 0.980 considering VMD, highlighting the enhanced potential for insulator failure detection in power systems.

Analyzing the audio signature of these motor failures can help correctly diagnose which component is at fault. In this paper, audio data were collected during normal operation and under controlled failure conditions of compression ignition engines of different vehicle models. An analysis of the audio signals is performed to identify the features that can be used to classify standard operations and specific faults based on ML approaches. The main contributions of this paper are summarized as follows:

- Experiments were performed on three different vehicle models, and original data were collected from audio signals containing normal and engine failure situations.
- A hybrid approach combining the wavelet packet transform, Markov blanket feature selection, RandOm convolutional kernel transform, tree-structured Parzen estimator for hyperparameters tuning combined with ten machine learning classifiers was proposed.
- Wavelet packet transform was used to extract features from the audio signals, providing a detailed analysis of the frequencies associated with mechanical failures.
- Audio signals were classified by comparing the performance of ten machine learning models using the tree-structured Parzen estimator to explore the hyperparameter space. Hold-out and *k*-fold cross-validation strategies were applied.
- The proposed hybrid approach evaluates multiple classifiers, highlighting that three models perform well. These models demonstrate balanced performance, making them suitable for engine fault diagnosis that is evaluated here.
- Results indicate that RF, GBM, and LightGBM are promising alternatives for diagnosing engine faults from acoustic signals.

The remainder of this paper is organized as follows: Section 2 briefly presents the literature on engine fault diagnosis based on audio signal processing. Section 3 introduces the audio data source. Section 4 explains the proposed method, considering the feature selection method, preprocessing, hypertuning, optimization methods, and the considered classifiers. Numerical experiments applying different setups on the proposed method are presented and discussed in Section 5. Lastly, Section 6 summarizes this paper and outlines future work.

## 2. Related Works

Artificial intelligence algorithms for classification may be applied to estimate engine diagnosis. It works by using machine and deep learning models to categorize and identify potential issues or conditions within an internal combustion engine based on input data. It is important to note that the success of engine diagnosis classification relies on the quality and representativeness of the training data, the choice of appropriate features, and the selection of an effective ML model. Additionally, domain knowledge and expertise play a crucial role in interpreting the results and refining the model for accurate and reliable engine fault diagnosis [17].

Usually, standard classification models face the following drawbacks: (i) weak interpretability; (ii) underfitting behavior, (iii) unbalanced dataset, (iv) the susceptibility to outliers, and (vi) the need for hyperparameter tuning. Comprehending these limitations is imperative for specialists to make intelligent decisions regarding the selection and execution of classification models, considering the distinctive features and difficulties of their datasets and applications. Studies in the automotive literature on audio classification differ in terms of the sensors types used to collect data [18], how features are extracted, and how classification approaches are used. In general, many frequency domain-based approaches use image processing techniques for classification, while time domain approaches can be combined with many approaches, such as ML, deep learning, and hybrid models.

In recent years, researchers have actively adopted a variety of machine learning techniques for diagnosing engine faults. Cruz-Peragon et al. [19] utilized instantaneous engine speed and an artificial neural network (ANN) to identify the faulty cylinder in cases of misfire or abnormal combustion. The results demonstrated accuracy in obtaining engine characteristics such as the cylinder pressure curve, fuel consumption, and ignition time, allowing for the successful isolation of the faulty cylinder. In a diverse traffic environment characteristic of India, George et al. [20] employed an ANN and *k*-NN to detect and categorize about 160 cars belonging to three categories—heavy, medium, and light—from an audio signal. Mel-Frequency Cepstral Coefficients (MFCCs) were extracted for the detection of regions around peaks. The average accuracy obtained was 73.42%. Alexandre et al. [21] proposed an application capable of classifying vehicles based on the sound they produce to keep traffic noise within tolerable levels for human health and improve intelligent transportation systems for short-term traffic flow forecasting. The classifier based on extreme machine learning and a genetic algorithm performs with an average classification probability of 93.74% when using the optimal subset of selected features.

Kerekes et al. [22] explored a multimodal detection technique using 14 characteristics, sensor data with electromagnetic emanations, and audio signatures for vehicle classification and identification. A supervised kernel regression method was used to classify and identify vehicles without the need for invasive images, with an average accuracy of 86% to 94%. Using frequency spectrum data from acoustic signals in wireless acoustic sensor networks, Ntalampiras [23] developed an acoustic classifier based on the echo state network for moving automobiles, achieving an average accuracy of 96.3%. To collect fault data without inducing failure in a real engine, Rubio et al. [24] constructed a diesel engine failure simulator based on a one-dimensional thermodynamic model, replicating engine behavior under failure conditions, with most of the simulated responses aligning closely with the experience database.

The behavior of drivers near control points was observed by Kubera et al. [25] to check whether driving is safe both when approaching the radar and after passing it. The data were classified into three classes: car acceleration, decelerating, or with constant speed. The SVM, RF, and ANNs were used as classifiers through time-series-based approaches. An ensemble classifier was proposed and achieved an accuracy of almost 95%.

Yiwere and Rhee [26] investigated the sound source distance estimate utilizing a convolutional recurrent neural network as an image classification task. After converting the audio signals into MFCC format, the classification model shows excellent classification accuracy, with 88.23%. Tran and Tsai [27] developed an automatic detection system to recognize emergency cars when their sirens sound, alerting other drivers to pay attention. With WaveNet and MLNet, a convolutional neural network (CNN)-based ensemble model was generated to identify sounds using a combination feature made from MFCC and log-mel spectrogram, with a 98.24% classification accuracy. An innovative method for defect diagnostics of automobile power seats using a smartphone was proposed by Huang et al. [28]. The *k*-NN and SVM models were utilized to identify faults with superiority. Recently, a 1D-CNN model with 98.37% accuracy was presented by Parineh et al. [29] for emergency vehicle identification.

Zhao et al. [30] introduced a novel diesel engine fault diagnosis method for multiple operating conditions. The researchers enhanced the condition adaptability of fault diagnosis by incorporating the Mel frequency transform and adaptive correlation threshold processing into vibrational mode decomposition and MFCC frameworks. Subsequently, they employed the *k*-NN for classification. Cai et al. [31] integrated a rule-based algorithm with Bayesian networks/back propagation neural networks for diagnosing faults across a broad spectrum of rotation speeds, utilizing training data derived from fixed speeds. Furthermore, they employed a novel data-driven diagnostic method based on Bayesian networks for diagnosing permanent magnet synchronous motor issues [32], demonstrating that the proposed methods exhibited effective diagnostic performance for early faults.

Stoumpos and Theotokatos [33] introduced a methodology that combined thermodynamic, functional control, and neural networks data-driven models for engine health management. The proposed method demonstrated the ability to capture engine sensor anomalies and make corrections. Kong et al. [34] conducted fault diagnosis studies on closed-loop control systems using dynamic Bayesian networks. Analyzing the studies presented previously, it is possible to highlight that ANNs have made considerable progress in the recent decade when applied to vehicle audio analysis. However, this literature review identifies research gaps that should be addressed. Common classification algorithms such as RF, *k*-NN, SVM, and neural networks are viable; however, they have advantages and disadvantages.

While the ANNs may have high accuracy, they struggle with prolonged learning time. Furthermore, the learning process of the neural network remains usually unobservable, and the resulting outputs are challenging to interpret, adversely affecting the credibility and acceptability of the results. The *k*-NN, categorized as lazy learning, demands extensive computation and lacks interpretability for classification results. The SVM, though needing fewer samples, primarily applies to two-class classification. However, diesel engine fault diagnosis requires multi-class classification, typically necessitating the combination of multiple two-class SVMs. In contrast, the RF can elucidate the classification process, support the theoretical analysis of diesel engine faults, and satisfy the requirements of multi-class classification for diesel engine faults. There is a trade-off between the complexity of the models and the computational processing cost [35]. Consequently, this study employs ten ML models for classifying the working state of the engine. Using an optimized hybrid method, the best combinations of models are employed.

The use of a suitable sensor can improve the quality of a measurement, thus improving the model’s performance results. A lot of technology has been presented to obtain the best possible measurement based on the latest technology sensors. According to Zhao et al. [36], the quality of data plays a crucial role in the accurate classification of faults in systems that rely on sound data. High-quality sensors capture a broader and more precise range of frequencies, enabling the detection of subtle anomalies or variations in sound patterns that may signal mechanical or operational faults.

In contrast, low-quality sensors may introduce noise, distort important sound frequencies, or fail to capture certain acoustic signatures, leading to inaccurate or incomplete data [37]. This can result in the misclassification of faults or missed detections, reducing the overall effectiveness of fault diagnosis systems. High-resolution sensors, therefore, provide more reliable data for advanced analysis approaches, such as ML models, improving the accuracy and robustness of fault classification.

Regarding fault classification in combustion motors, in [38], the analysis utilizes time-frequency signal processing techniques like the fast Fourier transform and short-time Fourier transform, combined with ML algorithms, to detect and classify faults such as scuffing in engine components. The hybrid approach enhances diagnostic accuracy and efficiency, offering robust solutions for real-time engine fault identification, as demonstrated by high-performance results.

An overview of the integration of ML models in diagnosing faults in mechanical systems is presented in [39]. This review highlights the advancements in ML, particularly focusing on how deep learning and transfer learning enhance fault detection. The authors identify the challenges posed by imbalanced data in industrial systems and propose solutions to improve diagnostic performance. The selection of the appropriate model for fault identification can be a difficult task, and several approaches have been proposed to improve the classification of faults in engines and monitor their condition. Kefalas et al. [40] proposed the use of GBM combined with the discrete wavelet transform. By using extreme gradient boosting, they obtained promising results.

When analyzing the methods that make up a hybrid method, it is not possible to evaluate an individual method and specify which is the best, i.e., there is no single method that is the best, but it is the combination of these methods that makes the performance of the proposed hybrid method superior to the individual methods. Thus, the suggested hybrid approach combines hyperparameter tuning, feature selection (Markov blanket), data processing using WPT, and ROCKET together with 10 machine learning techniques for audio classification. This combination ensures that the performance of the classifiers is maximized and, at the same time, allows the model to collect the audio information accurately. In this way, the main advantage of the hybrid method is the combination of complementary techniques that allow both adequate feature extraction and precise model tuning, which leads to better classification performance.

## 3. Description of the Multi-Classification Task

The raw audio signal was collected experimentally from three vehicle models, with two vehicles selected from each model, all with compression ignition engines. Data on the sound of vehicle engines were collected in 50 specialized mechanical workshops, in 100 samples. Considering that this study seeks to enable the reproduction of experiments by users of vehicles with possible engine failures, we decided to use a smartphone device for data collection, which had a directional microphone located in the lower part of the device, with a response frequency ranging from 20 Hz to 20 kHz, for recording audio. Audio signals were collected from the vehicles at two different speeds, at idle (800–1000 rpm) and full load (2500–3000 rpm). The rotations chosen to capture engine audio were influenced by three factors:

- (i) The first factor is related to the ease of the user being able to capture the vehicle’s audio even if they need to leave the vehicle in a safe condition without needing to be inside the vehicle;
- (ii) The second factor is related to one of the impediments caused by injector failure, the vehicle’s electronic control center, when it detects the loss of engine power, limits the number of rotations to ensure that there is no damage;
- (iii) The last factor, both rotations, was chosen to evaluate and understand the performance of the vehicle types.

**Figure 1:**
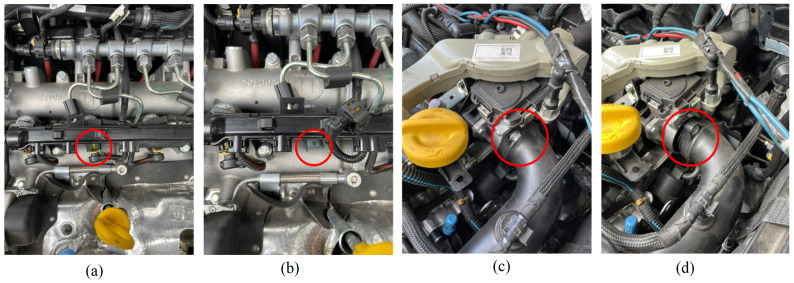
Example of simulated failures in the vehicles: (**a**) connected injector, (**b**) injector disconnected, (**c**) intake hose connected, and (**d**) intake hose disconnected.

The audio data collected contain ambient noise, which makes classification more challenging. To solve this issue, filters were used to reduce the non-linearities in the time series, as explained in the next section. For the selected vehicles, three different conditions were considered, such as the intake hose disconnected (class 1), one of the cylinders with the injector turned off (class 2), and no anomalies (class 3), as illustrated in Figure 1.

**Figure 2:**
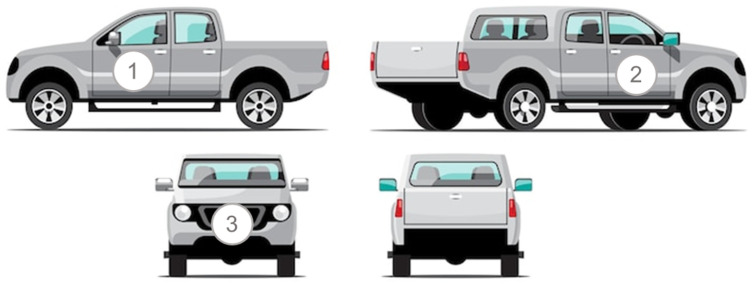
Audio data collection position of the vehicles.

Figure 2 presents the position of audio collection in vehicles, which was obtained in an external environment, in three different positions, two external, in front of the vehicle and next to the driver, and another internal. In the position of the vehicle driver, i.e., position (1) is the left or right side of the vehicle, position (2) in front of the vehicle, and position (3) inside the vehicle, front passenger. The recordings were made with a sampling rate of 22.05 kHz with files in the M4A format; for data compression, these files were converted to the WAV format.

**Figure 3:**
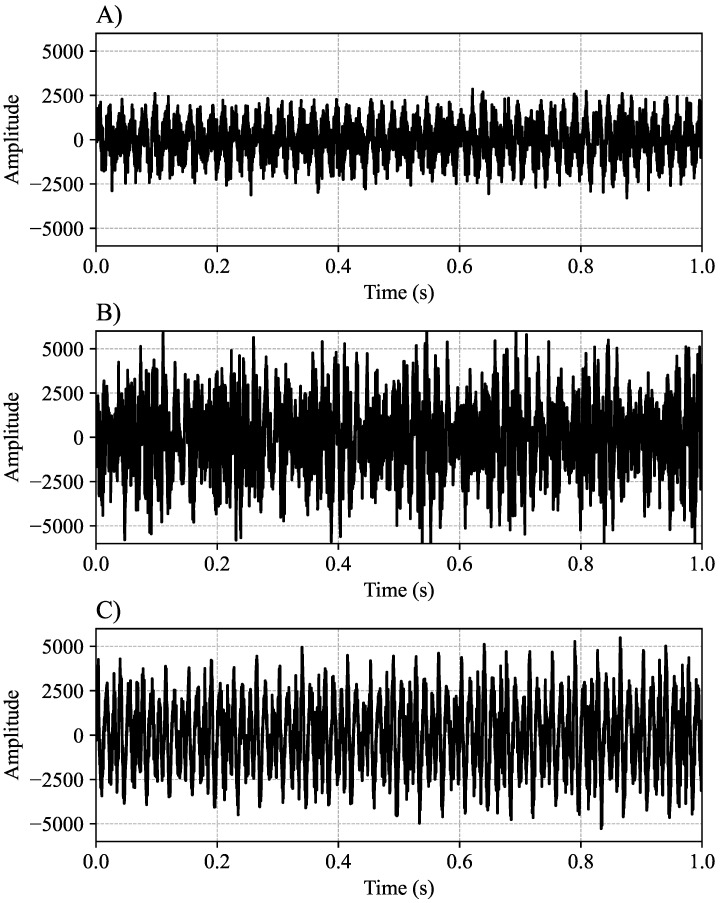
Original audio signal: (**A**) normal condition; (**B**) injector off; (**C**) air intake hose off.

Three different vehicle models were selected for data collection: two vans and a truck. To balance the classes, 550 s of each audio signal was segmented, and the dataset was normalized. Figure 3 shows the original audio signal with greater precision for normal conditions, injector off, and air intake hose off, respectively, showing one second of sampling; however, the audio was sampled for a total of 20 min.

The sound signal is represented in the temporal domain by one-dimensional data vectors (*X*),

(1) $$X=[x_{1},x_{2},x_{3},\ldots ,x_{n}],$$

where *n* is the total number of data samples in the dataset. Each data sample consists of a vector with length *l*, accompanied by a label $Y^{h}$, the classes, and can be represented as

(2) $$[\left(X^{1},y^{1}\right),\left(X^{2},y^{2}\right),\ldots ,\left(X^{n},y^{n}\right)],$$

where predicting the class label *Y* from the input vector *X* is the main goal of the proposed classification models.

The considered method is developed to be applied using a smartphone, which in the future could be embedded in an application to evaluate the motor condition based on the audio generated by it. The performance of this approach would be dependent on the quality of the device since low-quality microphones would imply poor performance, compared to high-quality microphones available on smartphones used currently. The quality of the microphone can introduce variability into the acoustics extracted from audio recordings. Microphones pick up varying ranges of sound frequencies with different levels of accuracy; sensitivity also determines how effectively the microphone picks up sound pressure levels. They also differ in terms of inherent noise generation, including self-noise and susceptibility to environmental noise. In this sense, smartphone microphones were used and measurements were made in a controlled environment and in triplicate to extract clear and reliable resources. The influence of microphone variability on classifier performance can be assessed using performance metrics—accuracy, precision, and recall—and it is possible to identify whether certain microphone types lead to a substantial degradation in classifier performance.

## 4. Methodology of the Proposed Approach

In this section, the fundamental concepts of each method that constitute the proposed approach, highlighting the drawbacks and potential benefits, are presented. The time series of the sound signal is filtered, as explained in Section 4.1, and the features are selected considering the Markov blanket principle and ROCKET (see Section 4.2). Using the relevant features, several classifier algorithms are applied. These classifiers are explained in Section 4.3. For these models, the hyperparameter optimization (explained in Section 4.4) using hold-out and five-fold cross-validation for training and validation splitting is used.

**Figure 4:**
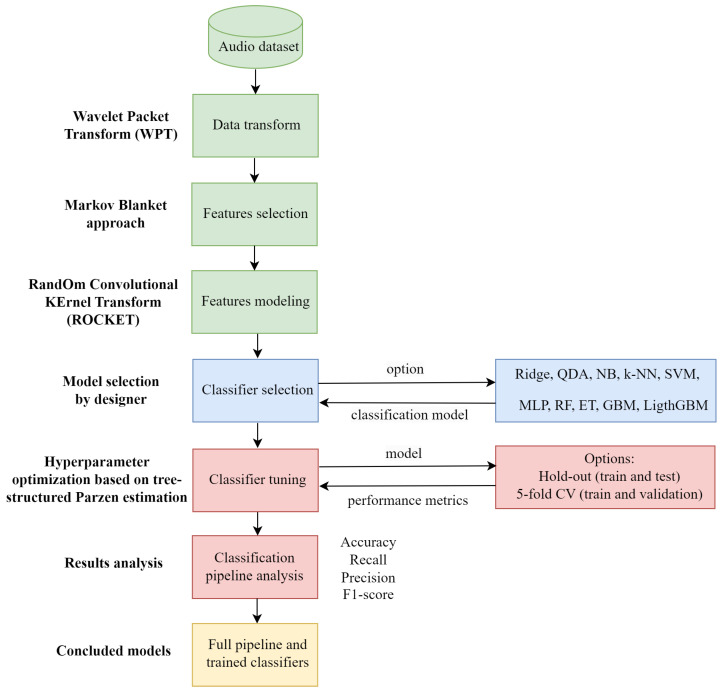
Flowchart of the proposed classification approach.

The flowchart of the proposed classification process is given in Figure 4.

### 4.1. Wavelet Packet Transform

A wavelet is a waveform of finite time with a null average value. The WPT is a multi-resolution signal analysis version of the standard wavelet transform. It is commonly used for dealing with non-linear and non-stationary time series. While the Fourier transform describes a signal as a sum of sines and cosines, the wavelet transform expands the original signal into a set (coefficient wavelets) of shifted and scaled versions of the base wavelet, known as the mother wavelet, which can represent data in both the time and frequency domains [41]. When it comes to producing additional frequency bands and improving the extraction of pertinent information from the original signal, WPT has been successfully employed to tackle engineering challenges. The number of coefficients is determined by the number of iterations that were completed since the WPT breaks down each iteration and extracts a coefficient. The decomposition of the wavelet obtains various levels of packet nodes, and from the wavelet packet, a tree structure is built by dividing the approximation of the coefficients.

### 4.2. Feature Selection

The Markov blanket is applied here to ensure that the most suitable signal features are selected. The Markov blanket principle, a wrapper-based method proposed in predictive permutation feature selection (PPFS), is used to select relevant features using the signals generated by WPT processing. Predictive permutation independence (PPI) is used to classify PPFS as a wrapper feature selection technique. The PPI test measures the degree of correlation between characteristics and the target variable using supervised algorithms. PPFS has both growth and shrink stages. In the growth phase, a candidate Markov blanket is conditioned on the empty set, and features are introduced by marginal independence. The shrinking phase that follows provides the optimal subset of features, which uses conditional independence to eliminate false positives among the candidate Markov blanket.

The process of finding a Markov blanket requires identifying conditional dependencies between features, which can become computationally intensive as the number of features increases. This is especially true in high-dimensional datasets where the search space for potential Markov blankets grows exponentially. With large datasets, especially those with many irrelevant or redundant features, the high dimensionality can make it difficult to effectively compute conditional dependencies. This may lead to overfitting or the inclusion of irrelevant features in the Markov blanket. For this reason, a cost–benefit assessment of its use is important when large datasets are considered.

#### RandOm Convolutional Kernel Transform

The ROCKET is a computationally efficient method for classifying time series compared to deep learning approaches. By using random convolutions, ROCKET reduces the complexity of feature extraction while maintaining classification accuracy [42]. It calculates features like the maximum value (Max) and the proportion of positive values (PPV) for each of the convolutional kernels. Given a time series $X=\{x_{1},x_{2},\ldots ,x_{n}\}$ and a convolutional kernel *K* with weights $w=\{w_{1},w_{2},\ldots ,w_{l}\}$ and bias *b*, the convolution result at position *i* is

(3) $$C_{i}=\sum_{j=1}^{l}w_{j}\cdot x_{i+j-1}+b$$

(4) $$\mathrm{MaxConv}\left(K,X\right)=\max\left(C_{1},C_{2},\ldots ,C_{n-l+1}\right)$$

(5) $$\mathrm{PPV}\left(K,X\right)=\frac{1}{n-l+1}\sum_{i=1}^{n-l+1}1\left(C_{i}>0\right).$$

**Figure 5:**
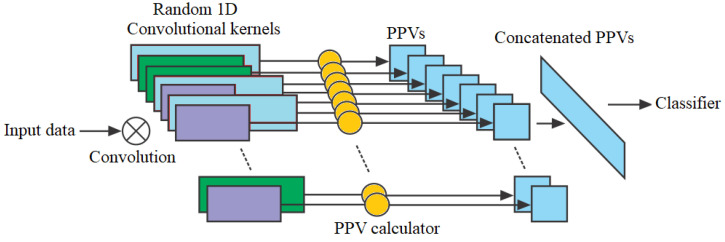
ROCKET architecture.

For *T* random kernels, the feature vector is

(6) $$F=[\mathrm{MaxConv}\left(K_{1},X\right),\mathrm{PPV}\left(K_{1},X\right),\ldots ,\mathrm{MaxConv}\left(K_{T},X\right),\mathrm{PPV}\left(K_{T},X\right)]$$

where *w* are the weights of the kernel of length *l*, *b* is the bias term (0 or 1), *n* is the length of the time series *X*, $C_{i}$ is the convolution result at position *i*, and 1(·) represents the indicator function. After that, a linear classifier for time-series classification is trained using the retrieved features [16]. The architecture of ROCKET is presented in Figure 5.

### 4.3. Classification Algorithms

To ensure that the best classification method is considered, this paper evaluates several ML-based algorithms. The considered classifiers are ridge regression, QDA, naive Bayes, *k*-NN, SVM, MLP, RF, ET, GBM, and LightGBM. These classifiers are explained in this subsection in detail.

#### 4.3.1. Ridge Regression

Ridge regression is a variant of linear regression in which a regularization factor (also known as a shrinkage or penalty term) is added to the usual linear regression cost function [43]. The ridge regression classifier minimizes a cost function that incorporates the sum of the features’ squared coefficients (weights) as well as the conventional classification loss. This regularization term reduces overfitting by penalizing significant weights, resulting in a smaller model that generalizes well to new, previously unseen data [44]. The cost function for the classifier using ridge regression is as follows:

(7) $$\mathrm{minimize}\left(\mathrm{Classification}\;\mathrm{Loss}+\alpha \sum_{i=1}^{p}w_{i}^{2}\right)$$

where $w_{i}$ are the weights of the features, and α is the regularization coefficient. Multicollinearity among features can impact the model’s stability in classification tasks. Classifiers using ridge regression are particularly useful when dealing with multicollinearity in the feature set, where features are highly correlated. The regularization term helps to mitigate the impact of multicollinearity.

#### 4.3.2. Quadratic Discriminant Analysis

The idea underlying QDA [45] is to model the distribution of features for each class and then apply Bayes’ theorem to compute the likelihood that an observation belongs to a specific class. The observation is subsequently assigned to the class that has the highest probability. QDA is a classification algorithm that uses distinct covariance matrices for each class, making it more adaptable in cases where the assumption of equal covariance matrices may not be valid. The QDA classification rule is given by

(8) $$P\left(Y=y|X=x\right)=\frac{p\left(y\right)\cdot f_{y}\left(x\right)}{\sum_{k}p\left(k\right)\cdot f_{k}\left(x\right)}$$

where P(Y=y|X=x) is the posterior probability of class *y* given the features *x*, p(y) is the prior probability of class *y*, and $f_{y}\left(x\right)$ is the probability density function of the features *x* given class *y*. The $f_{y}\left(x\right)$ is modeled using a multivariate normal distribution given by

(9) $$f_{y}\left(x\right)=\frac{1}{{\left(2\pi \right)}^{\gamma /2}\cdot {\left|\Sigma_{y}\right|}^{1/2}}\exp\left(-\frac{1}{2}{\left(x-\mu_{y}\right)}^{T}\Sigma_{y}^{-1}\left(x-\mu_{y}\right)\right)$$

where γ is the number of features, $\mu_{y}$ is the mean vector, and $\Sigma_{y}$ is the covariance matrix for class *y*.

#### 4.3.3. Naive Bayes

Naive Bayes is a classification algorithm based on Bayes’ theorem, a probability theory that associates an event’s likelihood with its prior knowledge. The naive refers to the assumption that the features used to describe instances are conditionally independent, given the class label [46]. The naive Bayes classifier frequently performs well in practice while remaining computationally economical. However, its accuracy may decrease if the independence assumption is severely broken in the data.

#### 4.3.4. *k*-Nearest Neighbors

Fix and Hodges [47] first developed the *k*-NN algorithm as a non-parametric supervised learning method, and Cover and Hart [48] later expanded it. The *k*-NN classifier is a non-parametric, instance-based technique that can perform both classification and regression tasks. During the training phase, the algorithm just stores the complete training dataset. When predicting a new data point, *k*-NN computes the distances between the new point and all other points in the training set, often using metrics such as Euclidean distance. It then uses these distances to identify the *k*-NN.

For classification tasks, the algorithm uses a majority voting scheme to assign the class label that is most commonly shared by these neighbors. Choosing the hyperparameter *k*, which represents the number of neighbors to examine, affects the model’s sensitivity to local fluctuations. One distinguishing feature of *k*-NN is its simplicity and absence of explicit training, making it simple to learn and apply. However, it may struggle with high-dimensional data and large datasets [49].

#### 4.3.5. Support Vector Machine

SVM has found widespread use in pattern recognition and text categorization. SVM achieves this by separating two data groups through hyperplanes or boundaries with the maximum margin of separation. The optimal separating hyperplane is the one that maximizes the distance between the hyperplane and the nearest points of both groups of data. SVM works especially well in classifying data points since it can identify the optimal hyperplane for dividing them into distinct classes in a high-dimensional environment. The aim is to find the decision boundary, or hyperplane, to maximize the margin between the classes [28]. The SVM is renowned for its proficiency in high-dimensional areas to manage intricate decision boundaries. The decision function of SVM can be expressed as

(10) $$f\left(x\right)=\mathrm{sign}\left(\sum_{i=1}^{N}\alpha_{i}y_{i}K\left(x,x_{i}\right)+b\right)$$

where *x* represents the input data point, *N* is the number of support vectors, $\alpha_{i}$ represents the Lagrange multipliers, $y_{i}$ represents the class labels of the support vectors, $K(x,x_{i})$ is the kernel function, and *b* is the bias term.

#### 4.3.6. Multilayer Perceptron

The MLP is composed of multiple layers of nodes that are connected. MLPs can be used to solve a variety of classification problems since they are efficient at modeling complex non-linear connections in data. To have non-linearity in MLP layers and allow the network to learn and describe complex patterns, non-linear activation functions (such as identity, logistic, tanh, and Rectified Linear Unit function) are utilized [50]. In this paper, the Adaptive Moment Estimation (Adam) optimization method is used with 1000 training epochs. The equations for a three-layer MLP are given by

(11) $$a_{j}^{\left(1\right)}=x_{j}\;\mathrm{for}\;j=1,2,\ldots ,n$$

(12) $$z_{i}^{\left(2\right)}=\sum_{j=1}^{n}w_{ij}^{\left(1\right)}a_{j}^{\left(1\right)}+b_{i}^{\left(1\right)}a_{i}^{\left(2\right)}=\sigma \left(z_{i}^{\left(2\right)}\right)\;\mathrm{for}\;i=1,2,\ldots ,m$$

(13) $$z_{k}^{\left(3\right)}=\sum_{i=1}^{m}w_{ki}^{\left(2\right)}a_{i}^{\left(2\right)}+b_{k}^{\left(2\right)}$$

(14) $$a_{k}^{\left(3\right)}=\mathrm{softmax}\left(z_{k}^{\left(3\right)}\right)\;\mathrm{for}\;k=1,2,\ldots ,K_{o}$$

where $a_{j}^{\left(1\right)}$ is the input to the hidden layer for feature *j*, $x_{j}$ is the input, $z_{i}^{\left(2\right)}$ is the weighted sum at the hidden layer node *i*, $w_{ij}^{\left(1\right)}$ is weight, $b_{i}^{\left(1\right)}$ is the bias, and $a_{i}^{\left(2\right)}$ is the output of hidden layers. The activation function at the hidden layer is represented by σ(·), $z_{k}^{\left(3\right)}$ is the weighted sum at the output layer node *k*, $w_{ki}^{\left(2\right)}$ is the weight from hidden layer node *i* to output layer node *k*, $b_{k}^{\left(2\right)}$ defines the bias at output layer node *k*, $a_{k}^{\left(3\right)}$ is the output of output layer node *k*, softmax(·) is the softmax activation function for classification, *n* is the number of input features, *m* is the number of nodes in the hidden layer, and $K_{o}$ is the number of classes in the output layer.

#### 4.3.7. Random Forest

Breiman [51] proposed RF as an ensemble ML method. It works by creating an ensemble of decision trees, each trained on different subsets of the data and features. During prediction, each tree in the forest provides a classification result, and the final output is determined by taking a majority vote for classification. This method is not limited to regression and classification but also exhibits excellent behavior in variable selection. *T* is the total number of trees in the forest, and its prediction for an input *x* is represented by T(x). The final forecast for a classification task (voting) in a RF with *N* trees can be expressed as follows:

(15) $$\hat{y}=\mathrm{mode}\left(T_{1}\left(x\right),T_{2}\left(x\right),\ldots ,T_{N}\left(x\right)\right)$$

where $\hat{y}$ is the predicted class, and mode represents the most frequently occurring class among the predictions of individual trees [52].

#### 4.3.8. Extra Trees

Extra Trees (ET) classifier is an ensemble learning method closely related to RF [53]. ET is a based ensemble method, but it differs significantly in how it builds individual trees. Adding more unpredictability to the tree-building process is the main notion behind ET. In an ET ensemble with *N* s, the final prediction for a classification task (voting) is given by

(16) $$\hat{y}=\mathrm{mode}\left(E_{1}\left(x\right),E_{2}\left(x\right),\ldots ,E_{N}\left(x\right)\right)$$

where $\hat{y}$ is the predicted class, and mode represents the most frequently occurring class among the predictions of the individual trees.

#### 4.3.9. Gradient Boosting Machine

Proposed by Friedman [54], the Gradient Boosting Machine (GBM) is an ensemble learning classifier from the boosting algorithm family. The GBM actively constructs an efficient classification or regression model through a sequential learning process. GBM generates a predictive model in the form of an ensemble of weak learners (usually s), gradually improving the model’s performance by reducing the errors of prior models.

The GBM starts by fitting a regression tree to the data, calculating predictions, and determining the initial residue. Subsequently, a new model is fitted to the previous residue, incorporating a new prediction, adding the initial forecast, and generating a new residue. This iterative process continues until a convergence criterion is met. Each iteration involves fitting a new model to the data to address the weaknesses of the previous model. The GBM can be expressed mathematically as follows:

(17) $$F\left(x\right)=F_{\mathrm{init}}\left(x\right)+\eta \sum_{m=1}^{M}\beta_{m}h_{m}\left(x\right)$$

where F(x) is the final prediction, $F_{\mathrm{init}}\left(x\right)$ is the initial model, η is the learning rate, *M* is the number of weak learners, $\beta_{m}$ is the contribution weight of the *m*-th weak learner, and $h_{m}\left(x\right)$ is the prediction of the *m*-th weak learner.

#### 4.3.10. Light Gradient Boosting Machine

To enhance calculation efficiency and partition the parameters of the input layer into distinct parts for establishing the mapping relationship between the input and output, the Light Gradient Boosting Machine (LightGBM) is used [55]. The LightGBM is intended for distributed and efficient training, which makes it ideal for huge datasets and challenging machine learning problems. To optimize a loss function, it adds weak learners to the model one after the other during training. The update equation for the final prediction after *M* weak learners is given by

(18) $$F_{M}\left(x\right)=F_{M-1}\left(x\right)+\gamma \cdot h_{M}\left(x\right)$$

where $F_{M-1}\left(x\right)$ is the prediction of the model after M−1 iterations, γ is the learning rate, and $h_{M}\left(x\right)$ is the prediction of the *M*-th weak learner. The weak learner at iteration *M* is trained to minimize the loss function L, where the negative gradient is used, as follows:

(19) $$h_{M}\left(x\right)=\arg\min_{h}\sum_{i=1}^{N}{\left[-\frac{\partial L(y_{i},F_{M-1}\left(x_{i}\right))}{\partial F_{M-1}\left(x_{i}\right)}\right]}^{2}+\Omega \left(h\right)$$

where $y_{i}$ is the true label for the *i*-th instance, $F_{M-1}\left(x_{i}\right)$ is the current prediction of the model, and Ω(h) represents a regularization term to control the complexity of the weak learner.

### 4.4. Hypertuning

Random search, grid search, metaheuristics [56,57], and Bayesian optimization [58] are some of the possible hyperparameter tuning methods. The Optuna based on TPE is used here to tune the hyperparameters of all models. It is a Bayesian optimization method. It is designed to explore the hyperparameter space by iteratively evaluating and refining the search. The TPE approach involves constructing probabilistic models to approximate the objective function and guide the search process. These models help to decide which hyperparameter configurations to evaluate next, based on the outcomes of previously tested configurations [59].

Hyperparameter space is a set of all possible hyperparameter configurations that need to be explored to find the optimal combination. TPE uses density estimators to model the distribution of the objective function values. TPE models the distribution of hyperparameter configurations using two density functions, the density of configurations with good performance (l(x)), and the density of configurations with poor performance (g(x)) [60]. The TPE calculates the expected improvement (EI) criterion to select the next set of hyperparameters to evaluate, calculated by EI(x)=l(x)/g(x).

### 4.5. Performance Indicators of the Classifiers

In this work, to compare the models in the multi-classification task, the accuracy, recall, precision, and F1-score are considered [46]. The accuracy is the ratio of correctly predicted instances to the total number of instances, given by

(20) $$\mathrm{accuracy}=\frac{\sum_{i=1}^{N}C_{ii}}{\sum_{i=1}^{N}\sum_{j=1}^{N}C_{ij}}$$

where *N* is the number of classes in the multi-classification problem, $C_{ii}$ is the count of instances of class *i* correctly predicted, and $C_{ij}$ is the count of instances of class *i* predicted as class *j*.

The indicator ${\mathrm{recall}}_{i}$ is the ratio of correctly predicted instances of class *i* to the total number of actual instances of class *i*. It can be calculated by the following expression:

(21) $${\mathrm{recall}}_{i}=\frac{C_{ii}}{\sum_{j=1}^{N}C_{ij}}\;\mathrm{for}\;i=1,2,\ldots ,N.$$

${\mathrm{precision}}_{i}$ is the ratio of correctly predicted instances of class *i* to the total number of predicted instances as class *i*, where

(22) $${\mathrm{precision}}_{i}=\frac{C_{ii}}{\sum_{j=1}^{N}C_{ji}}\;\mathrm{for}\;i=1,2,\ldots ,N.$$

F1-score_*i*_ for class *i* is the harmonic mean of precision and recall, given by

(23) $${F1\text{-}\mathrm{score}}_{i}=\frac{2\cdot {\mathrm{precision}}_{i}\cdot {\mathrm{recall}}_{i}}{{\mathrm{precision}}_{i}+{\mathrm{recall}}_{i}}\;\mathrm{for}\;i=1,2,\ldots ,N.$$

## 5. Results and Discussion

The experiments were computed using Google Colab, considering a central processing unit of 2.30 GHz with 2 cores and 12 GB of random access memory. The analysis was performed using the 32, 28, and 40 samples from the 100 samples that were obtained experimentally for classes 1, 2, and 3.

In terms of the audio signal transformations, the WPT generates 576 features. After, the feature selection based on the Markov blanket approach selects 56 predictive features. The selected features are employed in ROCKET, which utilizes 3000 kernels, balancing the trade-off between classification accuracy and processing efficiency. Dimension 2940 represented the output signal produced by ROCKET. This signal was fed into the ten ML classifiers that were evaluated using the ROCKET approach.

Table 1 presents the classification results for various classifiers used in the ROCKET structure with a dataset split of 90% for training and 10% for testing. In terms of accuracy, ROCKET, using the ridge model, presented a training accuracy of 100%, while the testing accuracy was 80%. This suggests a potential overfitting issue. QDA obtained both training and test accuracies that were relatively low, indicating that the model may not be capturing the underlying patterns in the data well. Other classification models including NB, *k*-NN, SVM, MLP, RF, ET, GBM, and LightGBM performed with high training and test accuracies, suggesting solid overall model performance.

Regarding recall, the ridge had high values for both training and test sets, indicating an effective ability to capture true positive instances. QDA presented low values, especially on the testing set. Possibly, the model might have been missing a significant number of positive instances. The other evaluated models exhibited generally high recall values, showing good performance in capturing true positive instances. Ridge demonstrated high precision on both training and test sets, signifying a minimal occurrence of false positives. QDA exhibited low precision values, particularly on the test set, implying a higher incidence of false positives. Other evaluated classification models showcased consistently high precision values, indicating a low frequency of false positives across these models.

Observing the F1-score, the ridge model achieved promising F1-score values, skillfully maintaining a harmonious equilibrium between precision and recall. QDA registered suboptimal F1-score values, signaling inadequate overall model performance in terms of precision and recall. The other models consistently demonstrated robust F1-score values, indicative of an equilibrium between precision and recall. The time indicates the time taken for each model to be trained. MLP has the highest training time, followed by LightGBM and RF. Other evaluated models have relatively lower training times. The fastest model was NB, requiring 27 s to be trained.

The three models perform well on the training dataset, achieving high accuracy and minimal misclassifications. On the testing set, these models maintain good accuracy, but there are some misclassifications. The ridge model has a few misclassifications in Class 1 during testing. The GBM and LightGBM models have no misclassifications in testing but have a small difference in predictions for Class 3. The LightGBM model has a slightly better performance on the testing dataset than the other models.

The search space used in hyperparameter tuning in all experiments of hold-out and *k*-fold cross-validation strategies via the Optuna framework using TPE with 300 trials is presented in Table 2. The best tuning of the hyperparameters using the Optuna TPE optimizer in combination with ROCKET for 90% training and 10% test splitting is presented in Table 3. The ridge, QDA, and NB presented relatively simple with a single hyperparameter being tuned. There was no great difference between the use of the cross-validation method in the classification assessment. In the variations of data splitting (90/10 and 70/30), cross-validation was not considered.

The *k*-NN had two hyperparameters tuned, suggesting a moderate level of complexity in controlling the number of neighbors and the leaf size of the tree. SVM shows a higher level of complexity with multiple hyperparameters tuned, indicating flexibility in kernel selection and regularization. MLP has three hyperparameters tuned, reflecting the architecture of an ANN with specified units, activation function, and regularization strength. RF, ET, GBM, and GBM had a considerable level of complexity with numerous hyperparameters tuned.

Classification results from multiple classifiers in the ROCKET framework are presented in Table 4, where the dataset is split 80% for training and 20% for testing. An analysis of the key performance metrics for each model showed that the ridge had high training accuracy but lower test accuracy (0.60), indicating potential overfitting. This also happened considering recall and precision. QDA obtained moderate accuracy for both training and test sets, and low precision for both sets, suggesting a higher rate of false positives. The F1-score was relatively low for the QDA too. NB exhibited moderate accuracy, with better performance on the training dataset. Balanced recall and precision for both sets were obtained with the NB approach.

Higher accuracy on the test set was achieved by the *k*-NN classifier as compared to the training set. Both sets exhibit high recall, but the test set provided more precision. The SVM presented excellent training accuracy but lower test accuracy, indicating possible overfitting, high precision, and recall for both sets. MLP seemed to be exhibiting overfitting behavior, evidenced by its high training accuracy but inadequate test accuracy. Moreover, MLP consistently showed F1-score, recall, and precision for both sets.

For the tree-structure models, RF behaved well on both datasets. Additionally, it revealed excellent results in the classification with good precision, recall, and F1-score. For all the training and test sets, ET achieved moderate accuracy with good precision, recall, and F1-score. The GBM produced a little lower test accuracy but high training accuracy. Additionally, for both sets, GBM exhibited outstanding recall, precision, and F1-score. On both sets, LightGBM achieved good accuracy along with consistent precision, recall, and F1-score. Models that include SVM and MLP have been found to require higher training times than other classification models in terms of their processing time.

Like ridge, the GBM model correctly predicts one instance of Class 2 but misclassifies others on the training data related to Classes 1 and 3. High adaptability to the testing data can be observed by all three models, with only a few misclassifications. If compared to ridge and GBM, the LightGBM model exhibited a slightly greater number of misclassifications. Table 5 presented the best hyperparameter tuning for 80% training and 20% test splitting using the Optuna TPE together with the ROCKET approach and ML classifiers. A model’s complexity needs to be taken into account when evaluating computing resources, training time, and overfitting risk.

Table 6 presents classification results from multiple classifiers in the ROCKET framework with a split of 70% for training and 30% for testing. The ridge model achieved an accuracy of 100% on the training set but dropped significantly to 0.57 on the test set. This suggests potential overfitting of the training data. QDA provides comparable results on test and training sets, suggesting a well-balanced model, but with poor performance. On the test set, naive Bayes’ accuracy decreased. On the training set, *k*-NN achieved perfect accuracy; however, on the test set, its accuracy drops to 0.57. The SVM obtained 100% accuracy for training; however, on the test set, it significantly fell. RF and ET showed balanced accuracy on both sets. GBM achieved perfect accuracy on the training set but dropped slightly on the test set. In general, GBM presented high accuracy but at the cost of longer training times. In relation to hyperparameter tuning, LightGBM revealed a comparable performance.

On training and testing datasets, the ridge model scored 100% on accuracy, precision, and recall, suggesting outstanding results. On training and testing datasets, GBM also scored well, reaching perfect accuracy as well as high precision and recall. LightGBM did well overall, displaying high accuracy, precision, and recall on the testing dataset in particular. Table 7 presents the best hyperparameter tuning for 70% training and 30% test splitting.

A cost–benefit assessment can help with the trade-off between computational efficiency and accuracy. The Markov blanket approach was useful in feature selection, reducing the features from 576 to 56, while retaining features that are significant to the model, reducing dimensionality, training time, memory usage, and increasing computational efficiency. As datasets grow, scalability becomes critical. Cost–benefit analysis based on the performance of classification models concerning training duration, validation duration, accuracy, and scalability is relevant. Table 1, Table 4, and Table 6 illustrated that while certain models, such as GBM and LightGBM, achieve high accuracy and F1-scores, their computational time varies significantly, especially as the dataset size shifts from a 90/10 to a 70/30 training/test split. High test accuracy is often attained by tree-based models such as GBM and LightGBM (up to 1.00 in Table 1 with a 90/10 data split). However, it is noted that as the size of the training data increases, the computation time also increases. For example, the training time of the GBM model increases from 97 to 122 s.

Models such as ridge and k-NN present lower computation time; however, such models often have difficulty in preserving performance. Additionally, high-performing models need more processing time as the test set size grows, emphasizing scalability as an essential challenge regarding handling bigger datasets. All data splits indicate comparatively quick processing times for computationally simpler models like ridge and naive Bayes. Even though these classification models are not as accurate as advanced tree-based ensemble learning models, such as GBM or LightGBM, they may be suitable in situations where efficiency is considered more important than accuracy.

Table 8 presents classification results for a stratified *k*-fold cross-validation strategy, with the mean (μ) and standard deviation (σ) reported for *k* = 5. Models with 100% accuracy, recall, precision, and F1-score included *k*-NN, SVM, GBM, and LightGBM. With highly accurate and balanced precision and recall, ridge, RF, and ET all work well. Lower performance can be observed with QDA, NB, and MLP, especially in terms of accuracy and recall values. When compared to GBM and MLP, SVM and ET have comparatively faster computation.

According to the analyses presented here, the best performance results were achieved by the RF, GBM, and LightGBM models. One of the reasons RF often outperforms other classifiers is due to their use of bagging. In bagging, multiple decision trees are trained on different random subsets of data, and their predictions are aggregated. This reduces variance and mitigates overfitting, which makes RF robust compared to individual decision trees that tend to overfit the data. GBMs build trees sequentially, with each tree trying to correct the errors of the previous one. This boosting process allows GBMs to focus on the difficult-to-predict samples, reducing the bias in the model [61]. LightGBM builds trees based on the gradients of the loss function. It efficiently handles large datasets by using a histogram-based approach, which speeds up training and reduces memory consumption. Unlike traditional gradient boosting methods that grow trees level-wise, LightGBM grows trees leaf-wise. This approach can lead to a more complex tree structure and can capture interactions better, potentially improving predictive performance [62].

## 6. Conclusions and Directions of Future Research

This paper presented a hybrid classification approach consisting of the combination of WPT, Markov blanket-based selection method, ROCKET, TPE for hyperparameter tuning, and ten classification models of ML. The proposed approach is a cost-effective replacement for standard diagnostic techniques based on audio emission signals from compression ignition engines for fault scenario analysis. The proposed classification approach has acceptable precision in various automotive operating scenarios, as indicated by the multi-classification results based on the cross-validation carried out to model different engine problem scenarios. In particular, various classification metrics revealed the classifiers’ generalization performance in ROCKET across different classifiers, especially using ridge regression, GBM, and LightGBM. Considering that 70% of the data were used for training, the GBM model had an accuracy of 0.83 in the test phase, showing that it was possible to achieve acceptable results even when using a fraction of the data set, with less data being utilized for training. Comparing the results between the data evaluations, when using 90% of the data, the results of the model concerning accuracy reached 1.00 for the GBM and LightGBM models, being reduced to splitting the data using less data to train the model. This shows that the best selection is to use 90% of the data for training. Future studies could be carried out using other types of sensors for predictive maintenance, carried out by dealerships authorized to service vehicles. For use by end consumers, the development of applications could help with the initial identification of possible faults, facilitating the definition of the maintenance to be carried out.

## Figures and Tables

**Table 1:** Classification results using dataset splitting of 90% for training set and 10% for test set.

Model	Accuracy		Recall		Precision		F1-Score		Time (s)
	**Train**	**Test**	**Train**	**Test**	**Train**	**Test**	**Train**	**Test**	
Ridge	**1.00**	0.80	**1.00**	0.81	**1.00**	0.88	**1.00**	0.81	44
QDA	0.36	0.40	0.30	0.33	0.22	0.16	0.24	0.23	42
NB	0.52	0.50	0.52	0.53	0.53	0.68	0.51	0.46	**27**
*k*-NN	0.76	0.90	0.75	0.89	0.76	0.92	0.75	0.90	37
SVM	0.97	0.90	0.96	0.89	0.97	0.93	0.97	0.90	31
MLP	0.90	0.90	0.88	0.89	0.91	0.92	0.90	0.90	680
RF	0.64	0.90	0.61	0.89	0.63	0.93	0.62	0.90	150
ET	0.60	0.70	0.55	0.67	0.43	0.55	0.50	0.60	46
GBM	0.99	**1.00**	0.99	**1.00**	0.99	**1.00**	0.99	**1.00**	97
LightGBM	0.99	**1.00**	0.99	**1.00**	0.99	**1.00**	0.99	**1.00**	290

The best results are shown in bold.

**Table 2:** Hyperparameter search space used for each model.

Model	Hyperparameter	Minimum	Maximum
Ridge	alpha	0.1	10.0
QDA	reg_param	0.0	1.0
NB	var_smoothing	1 $\times \;10^{-12}$	1 $\times \;10^{-1}$
*k*-NN	n_neighbors	1	30
SVM	C	1 $\times \;10^{-5}$	1 $\times \;10^{5}$
	gamma	1 $\times \;10^{-3}$	100
	kernel	options: linear, rbf, poly, sigmoid	
	degree	2	5
	coef0	−1.0	1.0
MLP	hidden_layer_sizes	5	30
	activation	options: identity, logistic, tanh, relu	
	alpha	0.01	0.05
RF	n_estimators	5	30
	max_depth	1	20
	min_samples_split	0.1	1.0
	min_samples_leaf	0.1	0.5
	max_features	options: sqrt, log2, None	
	bootstrap	options: True, False	
	criterion	options: gini, entropy	
ET	n_estimators	5	30
	max_depth	1	20
	min_samples_split	0.1	1.0
	min_samples_leaf	0.1	0.5
	max_features	options: sqrt, log2, None	
	bootstrap	options: True, False	
	ccp_alpha	0.0	0.5
GBM	subsample	0.55	1.0
	learning_rate	0.02	0.05
	max_depth	1	20
	n_estimators	5	30
	min_samples_split	2	10
	min_samples_leaf	1	10
LightGBM	subsample	0.55	1.0
	colsample_bytree	0.75	0.95
	learning_rate	0.02	0.05
	n_estimators	5	30
	min_child_samples	5	20
	reg_alpha	1 $\times \;10^{-8}$	10.0
	reg_lambda	1 $\times \;10^{-8}$	10.0
	alpha	0	5.0
	num_leaves	2	2^10^

**Table 3:** Best tuning of models’ hyperparameters for 90% training and 10% test splitting.

Model	Tuned Hyperparameters
Ridge	{’alpha’: 2.1369}
QDA	{’reg_param’: 0.5293}
NB	{’var_smoothing’: 4.8593 $\times \;10^{-12}$}
*k*-NN	{’n_neighbors’: 4, ’leaf_size’: 4}
SVM	{’C’: 0.0175, ’gamma’: 0.0063, ’kernel’: ’poly’, ’degree’: 5, ’coef0’: −0.4639}
MLP	{’n_units_0’: 27, ’activation’: ’tanh’, ’alpha’: 0.0372}
RF	{’n_estimators’: 30, ’max_depth’: 11, ’min_samples_split’: 0.3981,
	’min_samples_leaf’: 0.1418, ’max_features’: None, ’bootstrap’: False, ’criterion’: ’entropy’}
ET	{’subsample’: 0.6994, ’learning_rate’: 0.0372, ’max_depth’: 4, ’n_estimators’: 22,
	’min_samples_split’: 10, ’min_samples_leaf’: 8}
GBM	{’subsample’: 0.5934, ’learning_rate’: 0.0417, ’max_depth’: 15, ’n_estimators’: 26,
	’min_samples_split’: 5, ’min_samples_leaf’: 7}
LightGBM	{’subsample’: 0.6699, ’colsample_bytree’: 0.8530, ’learning_rate’: 0.0480, ’n_estimators’: 20,
	’min_child_samples’: 7, ’reg_alpha’: 0.0073, ’reg_lambda’: 2.5584, ’alpha’: 0.5401,
	’num_leaves’: 939}

**Table 4:** Classification results using dataset splitting of 80% for training and 20% for testing.

Model	Accuracy		Recall		Precision		F1-Score		Time (s)
	**Train**	**Test**	**Train**	**Test**	**Train**	**Test**	**Train**	**Test**	
Ridge	**1.00**	0.60	**1.00**	0.61	**1.00**	0.59	**1.00**	0.59	67
QDA	0.39	0.40	0.33	0.33	0.28	0.16	0.26	0.23	72
NB	0.45	0.35	0.43	0.38	0.45	0.42	0.43	0.33	**29**
*k*-NN	0.46	0.70	0.42	0.68	0.43	0.76	0.42	0.70	94
SVM	**1.00**	0.70	**1.00**	0.69	**1.00**	0.74	**1.00**	0.71	53
MLP	**1.00**	0.65	**1.00**	0.65	**1.00**	0.65	**1.00**	0.65	686
RF	0.86	0.75	0.85	0.74	0.87	0.82	0.86	0.75	48
ET	0.73	0.80	0.70	0.79	0.80	**0.88**	0.70	0.80	69
GBM	**1.00**	**0.85**	**1.00**	**0.86**	**1.00**	0.87	**1.00**	**0.85**	749
LightGBM	0.91	0.80	0.91	0.79	0.91	0.80	0.91	0.80	171

The best results are in bold.

**Table 5:** Best tuning of models’ hyperparameters for 80% training and 20% test splitting.

Model	Tuned Hyperparameters
Ridge	{’alpha’: 0.4271}
QDA	{’reg_param’: 0.3323}
NB	{’var_smoothing’: 1.1343 $\times \;10^{-10}$}
*k*-NN	{’n_neighbors’: 21, ’leaf_size’: 28}
SVM	{’C’: 36553.2031, ’gamma’: 0.0587, ’kernel’: ’rbf’, ’degree’: 5, ’coef0’: 0.3712}
MLP	{’n_units_0’: 25, ’activation’: ’tanh’, ’alpha’: 0.0120}
RF	{’n_estimators’: 13, ’max_depth’: 12, ’min_samples_split’: 0.1189,
	’min_samples_leaf’: 0.1208,’max_features’: ’sqrt’, ’bootstrap’: False, ’criterion’: ’gini’}
ET	{’n_estimators’: 29, ’max_depth’: 3, ’min_samples_split’: 0.5151,
	’min_samples_leaf’: 0.1368, ’max_features’: None, ’bootstrap’: False, ’ccp_alpha’: 0.0329}
GBM	{’subsample’: 0.8202, ’learning_rate’: 0.0319, ’max_depth’: 17, ’n_estimators’: 26,
	’min_samples_split’: 5, ’min_samples_leaf’: 2}
LightGBM	{’subsample’: 0.7969, ’colsample_bytree’: 0.8211, ’learning_rate’: 0.0466, ’n_estimators’: 26,
	’min_child_samples’: 15, ’reg_alpha’: 0.1610, ’reg_lambda’: 2.3560, ’alpha’: 1.5265,
	’num_leaves’: 207}

**Table 6:** Classification results using dataset splitting of 70% for training and 30% for testing.

Model	Accuracy		Recall		Precision		F1-Score		Time (s)
	**Train**	**Test**	**Train**	**Test**	**Train**	**Test**	**Train**	**Test**	
Ridge	**1.00**	0.57	1.0	0.56	**1.00**	0.61	1.0	0.57	64
QDA	0.36	0.40	0.30	0.33	0.24	0.16	0.25	0.23	56
NB	0.44	0.33	0.43	0.32	0.59	0.23	0.42	0.27	42
*k*-NN	**1.00**	0.57	**1.00**	0.55	**1.00**	0.62	**1.00**	0.57	41
SVM	**1.00**	0.60	**1.00**	0.58	**1.00**	0.69	**1.00**	0.59	40
MLP	0.96	0.60	0.95	0.56	0.96	0.58	0.96	0.55	102
RF	0.60	0.63	0.55	0.58	0.43	0.52	0.50	0.55	54
ET	0.69	0.63	0.66	0.57	0.69	0.46	0.66	0.53	**33**
GBM	**1.00**	**0.83**	**1.00**	**0.84**	**1.00**	**0.85**	**1.00**	**0.83**	122
LightGBM	0.67	0.80	0.63	0.77	0.76	**0.85**	0.62	0.79	188

The best results are shown in bold.

**Table 7:** Best tuning of models’ hyperparameters for 70% train and 30% test splitting.

Model	Tuned Hyperparameters
Ridge	{’alpha’: 0.1248}
QDA	{’reg_param’: 0.2418}
NB	{’var_smoothing’: 0.0988}
*k*-NN	{’n_neighbors’: 1, ’leaf_size’: 14}
SVM	{’C’: 33.8637, ’gamma’: 0.2581, ’kernel’: ’rbf’, ’degree’: 2, ’coef0’: 0.6842}
MLP	{’n_units_0’: 19, ’activation’: ’tanh’, ’alpha’: 0.0179}
RF	{’n_estimators’: 5, ’max_depth’: 4, ’min_samples_split’: 0.9267, ’min_samples_leaf’: 0.1345,
	’max_features’: None, ’bootstrap’: False, ’criterion’: ’entropy’}
ET	{’n_estimators’: 12, ’max_depth’: 9, ’min_samples_split’: 0.6673, ’min_samples_leaf’: 0.1000,
	’max_features’: ’sqrt’, ’bootstrap’: False, ’ccp_alpha’: 0.0085}
GBM	{’subsample’: 0.9038, ’learning_rate’: 0.0494, ’max_depth’: 5, ’n_estimators’: 16,
	’min_samples_split’: 3, ’min_samples_leaf’: 3}
LightGBM	{’subsample’: 0.6322, ’colsample_bytree’: 0.7550, ’learning_rate’: 0.0234, ’n_estimators’: 30,
	’min_child_samples’: 16, ’reg_alpha’: 2.8243, ’reg_lambda’: 2.3752$\times 10^{-7}$, ’alpha’: 2.1963,
	’num_leaves’: 13}

**Table 8:** Classification results using stratified *k*-fold cross-validation strategy.

Model	Accuracy		Recall		Precision		F1-Score		Time (s)
	μ	σ	μ	σ	μ	σ	μ	σ	
Ridge	0.87	0.03	0.87	0.03	0.86	0.03	0.86	0.03	153
QDA	0.45	0.05	0.30	0.08	0.40	0.05	0.35	0.07	171
NB	0.38	0.02	0.35	0.04	0.37	0.02	0.35	0.03	**68**
*k*-NN	**1.00**	**0.00**	**1.00**	**0.00**	**1.00**	**0.00**	**1.00**	**0.00**	97
SVM	0.97	0.01	0.97	0.01	0.97	0.01	0.97	0.01	78
MLP	0.73	0.06	0.73	0.06	0.71	0.07	0.72	0.06	1136
RF	0.82	0.04	0.82	0.04	0.81	0.05	0.81	0.05	114
ET	0.70	0.02	0.70	0.03	0.67	0.02	0.68	0.02	95
GBM	**1.00**	**0.00**	**1.00**	**0.00**	**1.00**	**0.00**	**1.00**	**0.00**	2513
LightGBM	**1.00**	**0.00**	**1.00**	**0.00**	**1.00**	**0.00**	**1.00**	**0.00**	718

The best results are shown in bold.

## Data Availability

Data can be provided upon request.
